# Promoting Family Engagement With Early Childhood Developmental Screening via the Baby Steps Text Messaging and Web Portal System: Longitudinal Randomized Controlled Trial

**DOI:** 10.2196/73443

**Published:** 2025-09-26

**Authors:** Hyewon Suh, Julie A Kientz

**Affiliations:** 1 Human Centered Design and Engineering, University of Washington, Sieg 428, Box 352315, Seattle, WA, 98195, United States, 1 2062210614

**Keywords:** developmental screening, human-centered design, text messaging, mHealth, patient engagement

## Abstract

**Background:**

Approximately 1 in 6 US children has a developmental disability. Early detection is crucial but often delayed, especially in families with limited access to resources. Current paper-based screening methods, such as the Ages and Stages Questionnaire, face challenges such as cultural barriers and timing issues. Digital tools can improve parent engagement and screening accuracy. This research explores new technologies to enhance long-term parent involvement in developmental screening.

**Objective:**

The study aims to understand whether features of a digital intervention specifically designed to engage parents in developmental screening are effective over a long-term period.

**Methods:**

Parents of children between 7 and 12 months old were recruited through flyers at clinics and libraries, mailing lists, and social media, and then they self-enrolled after eligibility screening. We conducted a randomized controlled trial with 139 families over 20 months, along with follow-up interviews and surveys. The intervention consisted of an interactive web portal that combined developmental and sentimental record-keeping, family-friendly visualizations, and the ability to answer screening questions via multiple modalities (eg, text messaging and web), without involvement of health care providers. The control condition consisted of a web-based portal with no specific engagement features, modeled after standard web-based developmental screening tools.

**Results:**

Overall, we enrolled 67 parents in the control group and 72 parents in the experimental group, for a total of 139 enrolled participants. Several parent engagement strategies we deployed in the experimental group were effective in increasing milestone questionnaire completion, with text messaging standing out as the most impactful and efficient, offering the highest return relative to the effort required for its development and implementation. Overall, the experimental group demonstrated a 44% higher average response rate compared to controls (*t*_125_=−3.32, *P*<.01). Participants in the experimental group submitted significantly more timely and valid responses, after text messaging was introduced (phase 2: 95% vs phase 1: 71%; *t*_107_=−4.44, *P*<.01), which is a critical factor for effective and timely tracking of child development. The experimental group participants responded to more questions on average (mean 127.60, SD 49.01) than those in the control group (*t*_70_=−7.23, *P*<.01) in phase 2 as well. In addition, study completion rates were significantly higher in the experimental group (83% vs 30%; *t*_119_=−8.40, *P*<.01), indicating greater long-term engagement. Sentimental record-keeping features showed promise but limited use, suggesting the need for integration with tools parents already use.

**Conclusions:**

This study demonstrates that a human-centered design approach for technology-based interventions can significantly enhance parent engagement and completion rates of developmental screening questionnaires. However, further research is needed with a larger sample to determine whether such features effectively prompt parents to seek early intervention services. Future studies should focus on engaging more diverse and underserved populations to validate these findings.

## Introduction

### Background

Approximately 1 in 6 children between the ages of 3 and 17 in the United States are diagnosed with a developmental disability, such as autism, attention-deficit hyperactivity disorder, or intellectual disabilities [1]. The earlier developmental delays are detected, the sooner early interventions can start, which can result in better outcomes for these children [2]. Unfortunately, approximately half of the children who eventually receive a diagnosis of developmental delay are not identified until they reach kindergarten in the United States [3], with lower-income populations being missed at a higher rate [4]. Regular screening of child development is one of the primary mechanisms for early detection of many types of developmental disabilities.

The current approach for screening for developmental progress asks parents or primary caregivers a series of questions about a child’s abilities in areas such as gross motor, communication, and problem-solving. For example, “does your baby say two-syllable sounds, such as ‘da-da’ or ‘ga-ga’?” or “can your child walk upstairs while holding onto a railing?” A popular screener tool, the Ages and Stages Questionnaire (ASQ), consists of 22 questionnaires with 30 questions each across the child’s first 5 years [5]. The screener is administered via paper or through a website, often at Well-Child Visits with the child health care providers, by childcare providers, through community-based services, or by parents self-monitoring their child’s development. There are some limitations to using milestone questionnaires for screening, such as questions not being culturally competent, language or literacy barriers, or the potential for parent anxiety when tracking their child’s development [6]. However, developmental questionnaires are currently the most effective method for screening according to the American Academy of Pediatrics [7].

A major challenge with paper-based or even web-based screener surveys is that it is often difficult to ensure that parents complete each survey, especially if families are not regularly attending Well-Child Visits or are not yet connected to a Medical Home [7]. Given that Well-Child Visits are months to up to a year apart depending on the child’s age, these visits may not be the best timing for conducting timely screens. In addition, when parents answer screener questions in a single sitting in a time- and resource-constrained setting, such as in their child health care provider’s waiting room, they may not have the opportunity to try each activity with their child, such as drawing shapes on a piece of paper or interacting with a mirror. Also, busy or overwhelmed parents may just not have the time or available cognitive load to use paper- and web-based systems [8].

Digital interventions that can help prompt parents to answer questions at more regular intervals have the potential to encourage more complete and accurate responses, better patient engagement [9], and parent awareness of their child’s activities. Because child development takes place in daily life outside of traditional clinical settings, parents’ and caregivers’ reports of the early signs of developmental delay are the most reliable way to track them [10]. Therefore, it is important to engage, inform, educate, and empower parents to track and assess their child’s development in easier ways to make developmental screening more accessible, accurate, and meaningful. Human-centered design researchers have designed interactive digital tools that can promote this engagement [11-16]. However, there are limitations with standalone software or mobile apps, such as requiring the parent to remember to open the app to answer questions or device compatibility. Even with proactive notifications or email reminders, parents may still not engage as easily and as often as they might otherwise. Because these screening tools are intended to be used over a long period (eg, up to 5 years), they require specifically considered methods to maintain parent engagement over the long term.

Researchers in the computing, health informatics, and medical fields have focused on the usability and feasibility of technology-based interventions designed for use between and outside of clinic visits. Mobile health (mHealth) interventions, in particular, have been introduced to support various health domains for children [15], such as:

1. Providing pregnant mothers with information about pregnancy and newborns [17-19]
2. Promoting healthy infant feeding for obesity prevention [20]
3. Sending infant vaccine reminders [21]
4. Delivering speech developmental education [22]
5. Developmental monitoring and screening [23,24]

However, the long-term impact of mHealth interventions is not well understood due to the limited study periods of deployment studies, and further research is needed to establish long-term effectiveness. For example, Stowell et al [25] highlighted that engaging in extended interactions with participants through formative work can prevent researchers from making premature judgments and conclusions. This approach allows for a deeper understanding of the populations and their contexts. They also emphasized that without prolonged and thorough formative work, researchers might design evaluations based on a superficial understanding of the community’s challenges, attitudes, and resources, which can impact health inequities.

In this research, we aimed to (1) develop and evaluate novel techniques for leveraging interactive technology to engage parents in the developmental screening process and (2) assess the immediate and long-term effects of the designed systems in fostering family engagement with early childhood developmental screening.

### Design of Baby Steps Web Portal and Text Messaging Intervention

Based on preliminary research with key stakeholders [8,11,23], we identified potential methods for engaging families in the developmental screening process that would be combined in a new digital intervention called Baby Steps. We hypothesized that 3 key design features would help increase parent engagement in the screening process: (1) engaging visualizations to help families interpret results and guide them to appropriate actions, such as completing developmental activities or connecting with early intervention for evaluations; (2) combining developmental screening with sentimental record-keeping, such as keeping track of memories and fun activities; and (3) multiple, integrated ways to complete screening questionnaires (eg, via web or text messaging). Our goal was to understand whether these engagement features would lead to increased screening completion rates and thus ultimately lead to more connections to early support services if needed.

**Figure 1.**
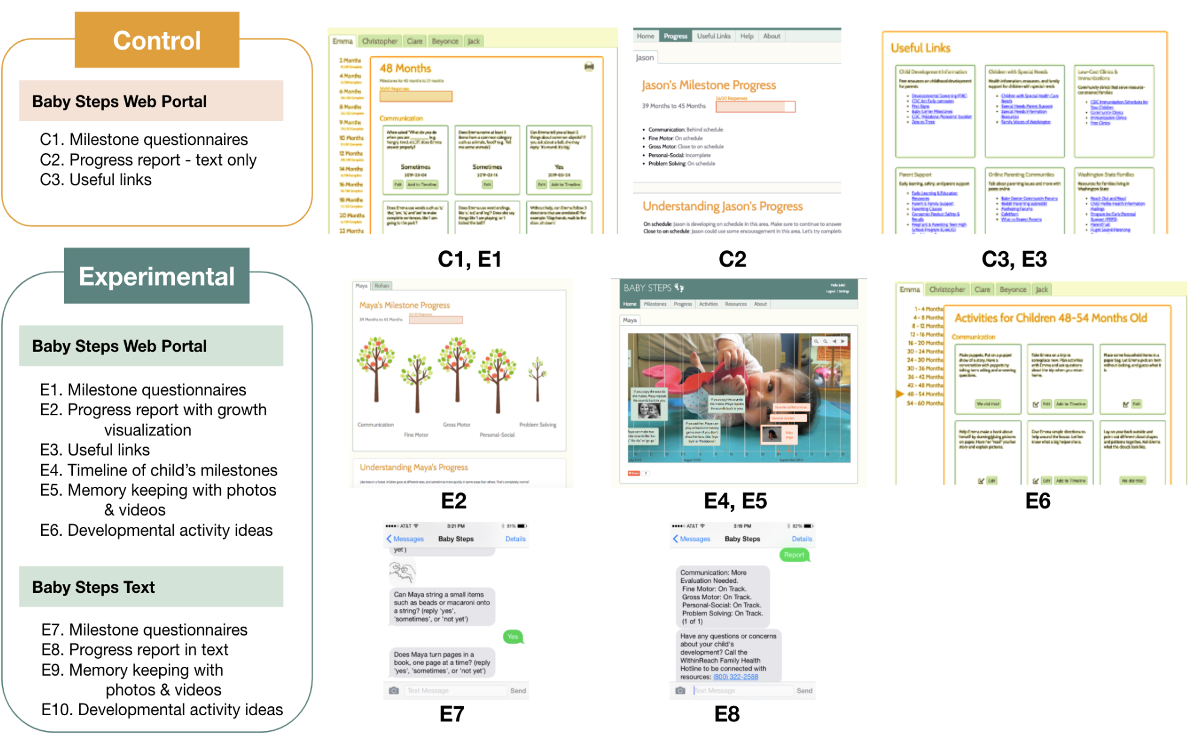
Baby Steps feature differences between control and experimental groups with screenshots. Photo of child included with parents’ consent.

We designed and implemented 2 versions of Baby Steps that would allow us to test these design features. We designed the control version of Baby Steps to be as close as possible to the current standard of care, which consists of neutral, web-based developmental screening questionnaires with text-based feedback on child progress. The experimental version of Baby Steps included additional features that tested our design hypotheses, such as tracking sentimental information and photos on the baby’s timeline, suggestions for activities that parents can do with their child to promote development, family-friendly visualizations of child’s progress that promote a growth mindset of development, and integration of text messaging prompts to complete milestone questionnaires that would synchronize with data on the web portal. Figure 1 shows the differences in features between the 2 versions, along with screenshots of those particular features. (The baby photo on the website screenshot was used with parental consent.)

## Methods

### Recruitment and Participants

We recruited parent participants via mailing lists, flyers posted in family-centered locations such as libraries, advertisements on social network services targeting parents of young children in Washington State, postings on local parenting lists, flyers at state-wide Women, Infants, and Children (WIC) clinics, and the University of Washington Communication Studies Participant Pool. We aimed to recruit parents who had a child between 7 and 12 months old, lived in Washington state, and were proficient in English to read and respond to milestone questions on the web portal. To ensure all participants meet the criteria, potential participants completed a screener survey. We recruited on a rolling basis, with the entire recruitment period taking about 7 months. To examine how specific design elements of the intervention affected self-monitoring behavior, we used a between-subjects design. A total of 180 participants completed the screener survey, and 139 were deemed eligible to enroll based on the age of the child and residing within Washington State. The first author used a random number generator to randomly assign participants to each group—if the generated number was odd, participants were assigned to the control group; if it was even, they were assigned to the experimental group. We then invited them to enroll in the study. Overall, we enrolled 67 parents in the control group and 72 parents in the experimental group, for a total of 139 enrolled participants.

### Study Procedure

Upon enrollment in the study, participants signed a web consent form and completed a prestudy survey on family demographics, child demographics, current parenting experience, and technology use. We then sent an invitation email to participants to create accounts and register on the Baby Steps web portal. The invitation email contained a brief introduction to the Baby Steps system, information about child development, and details on the study procedure, including the compensation plan. It also included an invitation code to use for portal registration, which was different for the control group and the experimental group. Depending on which invitation code was entered during registration, participants were exposed to 2 different web portal features as laid out in Figure 1. To study natural and realistic usage, we told our participants they could use Baby Steps as much or as little as they wanted, and we minimized interactions with the research team to allow for naturalistic use. Parents used Baby Steps to answer child developmental questionnaires, without involvement of health care providers. To encourage regular developmental screening, we included a paragraph noting to participants that if they did not interact with the Baby Steps system for more than 60 consecutive days, we might consider them to have withdrawn from the study and would send the exit package with a prorated incentive up to that point. However, we did not drop any participants until they reached the 12-month participation mark.

**Figure 2.**
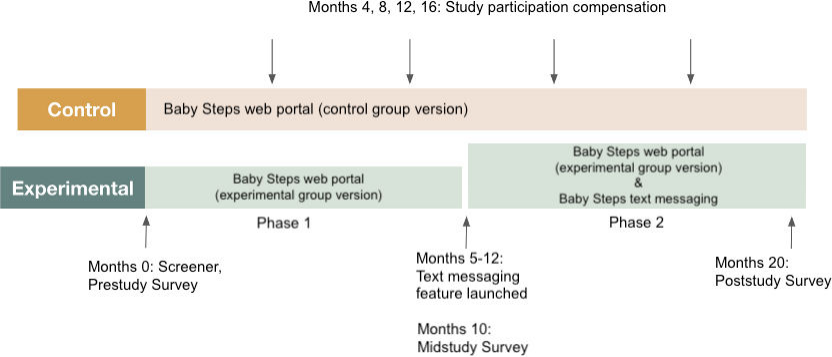
Overview and timeline of Baby Steps study.

To track Baby Steps usage, we instrumented the Baby Steps website with timestamped logging that allowed us to see which features were accessed by which participants and when. Due to some technical difficulties initially in the system development, the text messaging option for the experimental group could not be launched at the start of the study. Depending on when a participant started their participation, text messaging was introduced between 5 and 12 months into the study (Figure 2). When text messaging became available, the research team emailed the experimental group to introduce it and provided an instruction sheet. In total, 52 of the 72 experimental group participants registered their mobile phone numbers and began receiving milestone questions via text messages. This enabled us to also collect within-subjects data points from the experimental group and compare specific design characteristics of the web portal and text messaging. We will refer to the experimental group before text messaging as phase 1 and after text messaging as phase 2.

During the study, researchers only interacted with participants: (1) to send periodic compensations, (2) to introduce the text messaging option when it became available for the experimental group, and (3) to send a midstudy survey at 10 months. The midstudy survey was designed to receive participant feedback on their experience with Baby Steps, and 118 participants (54 in the control group and 64 in the experimental group) out of 139 enrolled participants completed the survey. At the end of the 20 months, we sent a poststudy survey to all 139 enrolled participants, regardless of how much of the study they had completed and received overall 87 responses (30 from the control group and 57 from the experimental group). Participants received a $20 Amazon.com gift card each at 4-, 8-, 12, 16-, and 20-month completion and a $10 Amazon.com gift card for each survey completion. In addition, participants who completed the entire 20-month study received a $20 Amazon.com gift card completion bonus. Participants who participated in a poststudy interview also received a $20 Amazon.com gift card.

We also partnered with WithinReach Washington [26], a local nonprofit organization and statewide Help Me Grow affiliate [27] that serves many low-income families and maintains a toll-free parent hotline number for parents to answer questions or address concerns about child development. The web portal’s progress report page directed parent participants to contact their child’s health care provider or the Help Me Grow Washington parent hotline for both the experimental and control conditions, as did the text message progress report in the experimental condition. We worked with WithinReach to cross-reference the names of study participants with parents who had registered with their system to analyze whether parents had reached out as a result of using either version of Baby Steps.

### Analysis

Data analysis focused on evaluating the impact of the Baby Steps intervention on parent engagement and the completion of developmental screening questionnaires over a long-term period. We used system usage logs and a survey to assess these. Given the importance of timely tracking of child development, we first separated responses based on whether parents completed milestone questions within the appropriate age range (responses within the valid time window vs those outside it). We then separated sources of response (website vs text messaging). Because the text messaging option was introduced later in the study, we were able to collect within-subjects data points for the experimental group (phase 1 vs phase 2) as well as between-subjects data (control group vs experimental group; and experimental group who used text messaging in phase 2 vs those who did not use). Descriptive analyses were used to analyze quantitative systems. Where appropriate, quantitative analysis was performed using *t* tests to compare the groups. For qualitative data from 3 surveys (intro, midstudy, and exit) and interviews, we conducted rapid qualitative analysis to identify key themes. These analyses aimed to determine the effectiveness of the design intervention in enhancing engagement and adherence to developmental screening protocols over the long term.

### Ethical Considerations

This study was reviewed and approved by the Institutional Review Board of the University of Washington (IRB 49545). All participants gave informed electronic consent before enrolling in the study and were informed about their right to opt out at any time. Participants received a US $20 Amazon.com gift card each at 4-, 8-, 12, 16-, and 20-month completion and a US $10 Amazon.com gift card for each survey completion. In addition, participants who completed the entire 20-month study received a US $20 Amazon.com gift card completion bonus. Participants who participated in a poststudy interview also received a US $20 Amazon.com gift card.

## Results

### Participant Demographics

**Figure 3.**
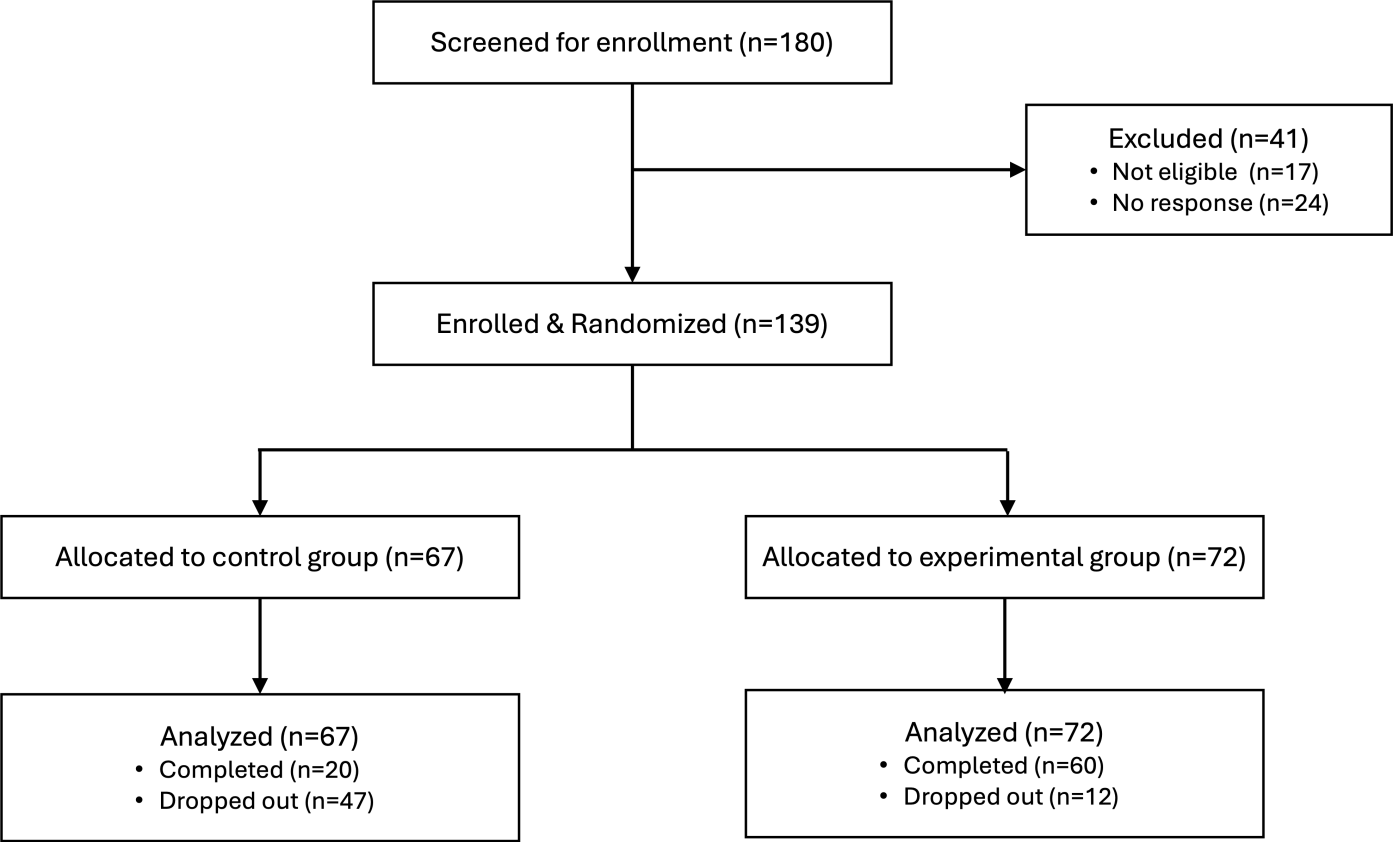
CONSORT (Consolidated Standards of Reporting Trials) flow diagram for the study.

Table 1 shows primary parent participant demographics across both groups. Despite our statewide recruitment efforts, the majority of the participants were from the greater Seattle, Washington metropolitan area. Our sample also overrepresents mothers and individuals from higher socioeconomic status compared to the general Washington State population (eg, education and income) [28], though it is closer to the average demographics for Seattle, WA, where the median household income is $118,745 and 38.1% have bachelor’s degrees and 31.9% have graduate degrees [29]. The sample does underrepresent the racial and ethnic diversity of the state as well as Seattle, especially that of Black, Hispanic, American Indian or Alaska Native, and Native Hawaiian or Pacific Islander families. This may have been a result of our lack of resources to provide the intervention and study materials in languages other than English. The complete participant flowchart is depicted in Figure 3.

**Table 1.** Participant demographics (N=139).

Characteristics	Number of participants, n (%)
Relationship to child	
Mother	134 (96.40)
Father	5 (3.59)
Number of children in family	
1	84 (60.43)
2	43 (30.94)
3	1 (0.72)
4 or more	11 (7.91)
Age of primary parent	
18‐24	8 (5.76)
25‐30	35 (25.18)
31‐40	87 (62.59)
41‐50	9 (6.47)
Marital status	
Married or partnered	131 (94.24)
Divorced or separated	2 (1.44)
Single, never married	5 (3.60)
Engaged	1 (0.72)
Education level of primary parent	
Less than 12th grade	1 (0.72)
High school or high school equivalency credential	4 (2.88)
Some college	17 (12.23)
College degree	58 (41.73)
Graduate degree	59 (42.45)
Parent Race or Ethnicity	
White	116 (74.84)
Asian	12 (7.74)
Hispanic or Latino	10 (6.45)
Black or African American	3 (1.94)
Other	5 (3.23)
Prefer not to say	9 (5.81)
Household income	
Less than US $15,000	5 (3.60)
US $15,000-$34,999	5 (3.60)
US $35,000-$49,000	10 (7.19)
US $50,000-$74,999	25 (17.99)
US $75,000-$99,999	22 (15.83)
Greater than US $100,000	61 (43.88)
Prefer not to say	11 (7.91)

### Developmental Screening Questionnaire Responses

There was a statistically significant difference between 2 groups in terms of screening questionnaire response rates (*t*_125_=−3.32, *P*<.01, 2-tailed). The experiment group yielded a total of 15,619 responses (mean 216.93, SD 102.42, median 205, [IQR] 145.75-306) compared to the control group’s 10,075 responses (mean 150.37, SD 130.95, median 90, [IQR] = 30-240), resulting in 44% more responses per participant on average. In addition, while there was no control group participant who updated their answers to questions (eg, changing “not yet” to “sometimes” or “yes”) when their child became able to meet the milestone, 20/72 (28%) experimental group participants updated their answers to questions, and 95% of those updates were made within the valid time range, indicating the experimental group participants were more engaged in monitoring their child’s development beyond answering milestone questions just once.

**Figure 4.**
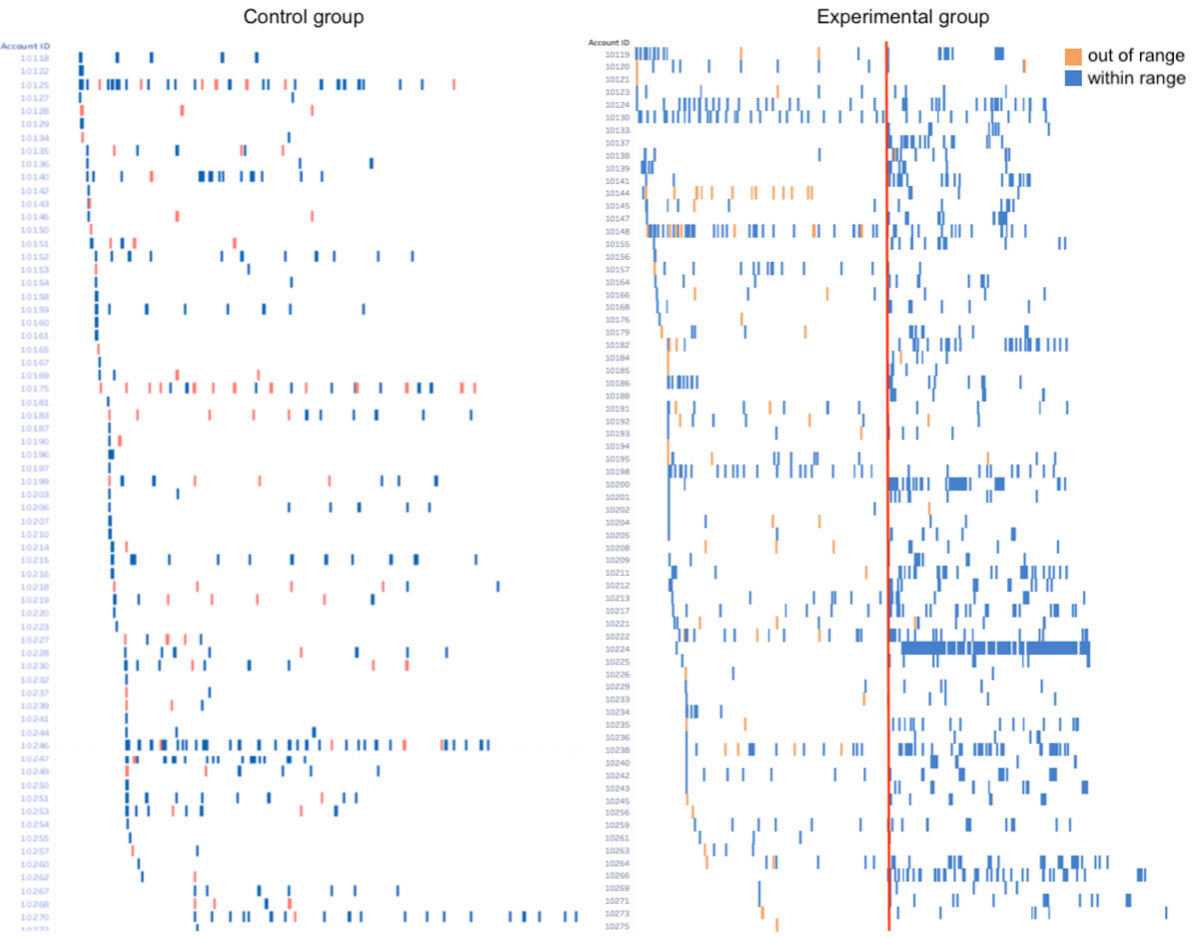
Chromograms of responses to developmental questionnaires over the entire study period. Blue lines indicate questions completed within the correct time range; orange lines indicate questionnaires completed outside of the recommended time range.

Figure 4 shows chromograms of responses to developmental questionnaires over the study period. Chromograms are a visualization technique used to detect and present patterns in large datasets [30]. This allows us to show the milestone response patterns of participants in each group over 20 months in one figure. In the figure, the left side represents the control group, and the right side represents the experimental group. Within each side, each participant is a row, and each column represents a day of the study. Because each questionnaire of ASQ is designed for a specific age range, answering outside of the valid age range may not accurately reflect a child’s development. We used blue to indicate responses recorded within the valid time range and orange to indicate responses recorded outside the valid time range (eg, the 9-month-old questionnaire was completed after the child was already 12 months old). The date at which text messaging was introduced for the experimental group is highlighted with a red line.

### Follow-Up With Providers Based on Screening Results

Another goal of this longitudinal study was to determine whether the experimental version of Baby Steps could nudge parents to follow up with child health care providers or early intervention services when the progress report indicated that more evaluation may be needed. The experimental group checked the web-based progress report 1.3 times more often than the control group (8.14 times per participant for the control group vs 10.46 times per participant in the experimental group) but this difference was not statistically significant. In addition, when we cross-referenced the list of study participants with the names of parents who had contacted WithinReach, there were only 6 total study participants that matched names with the WithinReach database (3 from the control group and 3 from the experimental group), and their contact had happened prior to the study, indicating that there was no participant who contacted WithinReach with concerns as a result of using Baby Steps. However, we did learn from our qualitative survey and interview responses that several participants in the experimental group contacted their child’s health care providers after reviewing the progress reports in Baby Steps.

*“Used this data to discuss delays with pediatrician, got referrals to early intervention services for therapy and other developmental delays and child will be starting developmental preschool next month with an IEP.”*

(E20)

*“On one occasion, he was delayed in his development (based on the progressreport) and at his doctor’s well check appointment I brought it up and after some tests, it was discovered that my child had iron deficiency. He was prescribed a supplement and right after, my son’s condition improved, and he eventually caught up to his development milestone. I would not have discovered or noticed the iron deficiency if not for the app”*

(E34)

### Engagement With Sentimental Memory Tracking and Timeline

We hypothesized that adding sentimental memory keeping to developmental tracking would help engage parents in a strength-based approach [31] to development rather than a deficit-focused one by allowing them to celebrate meaningful events in their child’s development (eg, first steps, first zoo visit, etc), which could help reduce emotional burden and promote positivity and resilience. However, memory keeping was not frequently used by the participants. Only 44/72 (61%) experimental group parents logged memories at least once, and of those, 23 (32%) used this feature 3 times or fewer. In the poststudy survey and interview, participants expressed uncertainty about keeping their memory logging data in the long term as a barrier to the usage of the feature.

*“I was not clear on how the information was going to be retained. At first, I was using the site every few days to track new developments, but then I realized that I probably wasn’t going to be able to keep the data. There was no option for printing it or saving it, so I ended up transitioning to a paper baby book instead.”*

(E27)

However, several participants in the control group suggested adding a memory-keeping feature to the Baby Steps system, implying a desire for combining developmental tracking with sentimental memory keeping.

*“(I want a) place to save photos of them [a child] attempting the tasks”*

(C26)

*“It would be nice to have a record of when my child was able to do some of the tasks at each milestone”*

(C3)

### Engagement With Web Portal Versus Text Messaging for Questionnaire Completion

#### Between-Subject Comparisons: Control Group Versus Experimental Group

While there was no statistically significant difference in the milestone question response rate between the control group and experimental group rate in phase 1, there was a statistically significant difference between the groups in phase 2, when 88% of the experimental group responses were from text messaging (*t*_136_=−7.71, *P*<.01). Five experimental group participants who answered fewer than 5 milestone questions via the website over 10 months of participation answered over 100 milestone questions respectively after text messaging was added in (in less than 10 months). This indicates that the introduction of text messaging significantly increased long-term participant engagement in the experimental group.

Also, before the text messaging option was introduced, the experimental group answered more questions within the valid time window than the control group (86% of the experimental group’s responses vs 72% of the control group’s responses) but the difference between the groups was not statistically significant. However, since the introduction of the text messaging option, the experimental group responses were statistically significantly more within a valid range (*t*_135_=−7.90, *P*<.01). Thus, the addition of text messaging improved the timeliness and validity of responses in the experimental group.

#### Between-Subjects Comparison: Experimental Group With Text Messaging Versus Without

The positive effect of using text messaging was evident again when we compared the experimental group who used text messaging in phase 2 (n=52) versus the experimental group who did not use text messaging in phase 2 (n=20). There was a statistically significant difference between the 2 groups (*t*_70_=−7.23, *P*<.01). The group that used text messaging answered 127.60 questions on average (SD 49.01, median 120 (IQR) 90.75-150 ), while the group that did not use text messaging answered 36.85 questions on average (SD 44.07, median 15.5 (IQR) 0-67.25 ), resulting in 246.27% more responses per participant on average. These findings underscore the substantial impact of text messaging on increasing participant response rates over time.

#### Within-Subjects Comparison (Phase 1 vs Phase 2)

In addition to between-subjects differences, when we compared within-subjects data of experimental group participants in phase 1 versus phase 2, the experimental group participants answered more milestone questions within the valid range in phase 2 (95%, 7279/7639) compared to phase 1 (71%, 5691/7980); *t*_107_=−4.44, *P*<.01. This within-subjects comparison further confirms the effectiveness of text messaging in enhancing response validity over time.

Overall, these findings demonstrate that the introduction of the text messaging option significantly enhanced long-term adherence among participants. This method not only increased the number of milestone questions answered but also improved the timeliness and validity of responses. Consequently, text messaging proved to be an effective tool for more accurate and timely tracking of child development, as reconfirmed by the positive feedback in the survey responses.

*“LOVED the text portion. It’s so easy to get side tracked with kids and constantly forget. I needed to text prompts and it made it easy to participate.”*

(E23)

*“Text was much much better, so easy to use and good for on the go. Gave me fun ideas to try in the moment as well.”*

(E45)

#### Overall Engagement and Completion of the Study

Finally, we ran descriptive statistics and compared general usage patterns between the 2 groups, and there was a significant difference in study completion rates. While only 30% (20/67) of the control group finished the entire 20-month study, 83% (60/72) of the experimental group remained enrolled in the study through the end (*t*_119_=−8.40, *P*<.01, 2-tailed), suggesting the experimental group study design was successful in engaging parents over a long period of time.

## Discussion

### Principal Findings

In this research, we aimed to (1) design and evaluate new approaches that use interactive technology to involve parents in the developmental screening process and (2) examine long-term impacts of these systems on promoting family engagement with early childhood developmental screening. Overall, design features to explicitly engage parents over time implemented in Baby Steps increased engagement and screening questionnaire completion rates. As early child developmental screening requires continuous monitoring over the first 5 years of a child’s life, it is promising that a large number of the experimental group participants were able to complete the 20-month study. However, the study was inconclusive regarding whether greater engagement led to connections to early intervention resources, and thus more research may be needed to understand what design features, if any, might prompt parents to follow up on making those connections. Because there were a very small number of cases, it is premature to conclude whether the differences in progress reports (text vs visualization) made a difference in follow-ups, but it is promising to know the progress reports were successful in triggering follow-up actions with their child’s health care providers for several parents.

The introduction of text messaging seemed to be a clearer success. We acknowledge that the proactive notifications that drew people to engage with the system were a big part of this, which prior research has also identified as important [32,33], and we also believe this success is due to the integration into a communication mechanism that is frequently used by parents already, which has been advocated for as a way to lower barriers to access [34] and can increase accessibility for lower-income populations without access to higher-end digital devices and data plans [35,36]. We believe that being able to answer milestone questions directly via text message, as opposed to just text-based reminders to log into the website, was a useful implementation of this feature that would benefit from further exploration.

More research is also needed to better understand the role that combining developmental screening with sentimental memory keeping can play in parent engagement. Although we saw low use of this feature overall, numerous parents reported finding the idea to be promising. However, because Baby Steps was separate from other ways that they tracked their baby’s sentimental milestones in baby books or using social media, the feature could be improved to better integrate with families’ needs. Adding the option to print information as physical books or integrate with Facebook or Instagram, which parents already frequently use to share information about their child with others [37] and benefit from [38,39], could be a promising next step.

### Limitations

Our study shows promising results for improving parent engagement with developmental screening, but this study is not without its limitations. Despite our best efforts for a broad recruitment approach, most of our study’s participants were of a higher socioeconomic status and less racially or ethnically diverse than the general population of Washington State [28]. It is known that many resource-constrained populations, such as low-income or immigrant families, lack the insurance to cover Well-Child Visits, frequently move, or face language barriers that make it difficult to access information about normal child development, and they often struggle because they may not be aware of what to monitor [40,41]. Therefore, further research is needed to examine the efficacy of interventions and monitor outcomes in larger, more diverse populations. As a follow-up to this work, the research team has started work via a community-based participatory research approach [42] to ensure that both technology designs and study designs better fit the needs of people from marginalized communities and to better understand how systems such as Baby Steps can fit into a broader macrosystem of care [43], including pediatric practices, community organizations, childcare providers, and public health organizations. Another limitation is that the ASQ currently has a cost per screen associated with it that we paid as part of our study’s grant. Future work could use an open-source screening tool, such as the Survey of Wellbeing in Young Children [44].

### Conclusions

This paper describes the findings from a long-term, randomized controlled trial of a novel technology-based intervention aimed to engage parents of children aged 0‐5 in the process of developmental screening. We enrolled 139 parent participants over a 20-month period and randomly assigned parents to use one of 2 versions of the system: one being a neutral, web-based screening questionnaire tool that mimics current standards for web-based developmental screening, and one that followed a human-centered design process to develop features aimed to engage parents in developmental screening over the long term. Our study determined that the version of the system designed via a human-centered process resulted in twice as many people completing the full study and engaged parents significantly more in completing timely developmental screening questionnaires. However, it was inconclusive whether the designed features would prompt parents to take actions such as engaging in activities to promote development or engaging with early intervention services if needed, as the overall number of children in the study who fell below the cutoff for developmental concerns was small. This study provides evidence that human-centered design methods for designing technology-based interventions can lead to systems that are engaging and improve the completion of developmental screening questionnaires. More community-based design research is needed to understand how to better engage families from more underserved communities.

## Supplementary material

10.2196/73443 Checklist 1 CONSORT-EHEALTH (V 1.6.1) checklist.

## Abbreviations

ASQ: ages and stages questionnaires
CONSORT: Consolidated Standards of Reporting Trials
JMIR: Journal of Medical Internet Research
mHealth: mobile health
WIC: women, infants, and children
